# Usability, Feasibility, and Effect of a Biocueing Intervention in Addition to a Moderated Digital Social Therapy-Platform in Young People With Emerging Mental Health Problems: A Mixed-Method Approach

**DOI:** 10.3389/fpsyt.2022.871813

**Published:** 2022-05-25

**Authors:** Marilon van Doorn, Laurens A. Nijhuis, Anne Monsanto, Thérèse van Amelsvoort, Arne Popma, Monique W. M. Jaspers, Matthijs L. Noordzij, Ferko G. Öry, Mario Alvarez-Jimenez, Dorien H. Nieman

**Affiliations:** ^1^Amsterdam University Medical Centers, Amsterdam, Netherlands; ^2^Department of Psychiatry and Neuropsychology, Maastricht University, Maastricht, Netherlands; ^3^Department of Medical Informatics, Amsterdam Public Health Research Institute, Amsterdam UMC-Location AMC, University of Amsterdam, Amsterdam, Netherlands; ^4^Department of Psychology, Health and Technology, University of Twente, Enschede, Netherlands; ^5^Buurtzorg Jong, Almelo, Netherlands; ^6^Centre for Youth Mental Health, The University of Melbourne, Parkville, VIC, Australia; ^7^Orygen, Parkville, VIC, Australia

**Keywords:** biocueing, indicative prevention, stress- and emotion regulation, youth mental health, e-health, early detection and intervention

## Abstract

**Introduction:**

To optimize treatment, it is of utmost importance to take into account the myriad of biological, social, and psychological changes that young people go through during adolescence which make them more vulnerable for developing mental health problems. Biocueing, a non-invasive method to transform physiological parameters into an observable signal, could strengthen stress- and emotion regulation by cueing physiologically unusual values in daily life. The aim of this study is to investigate the usability, feasibility, and exploratory effect of biocueing in addition to ENgage YOung people earlY (ENYOY), a moderated digital social therapy-platform, in young people with emerging mental health complaints.

**Methods:**

A user-centered mixed-method design was used. A focus group was conducted to optimize the ENYOY-platform and biocueing intervention. Biocueing was operationalized by a smartwatch and the Sense-IT app. A within-subjects design was used; 10 days for all participants ‘biofeedback off' (control), followed by 10 days ‘biofeedback on' (experimental). Emotional awareness and perceived stress were measured using ecological momentary assessment. Eight individuals participated. User-friendliness, usability, and acceptance were assessed using a qualitative design.

**Results:**

Findings from the focus group resulted in several adaptations of the biocueing intervention to the ENYOY-platform and vice versa. The average measurement compliance rate was 78.8%. Level-one findings showed different individual effects on perceived stress and emotional awareness. Level-two analyses showed no overall effects on perceived stress (*B* = −0.020, *p* = 0.562) and overall positive effects on emotional awareness (*B* = 0.030, *p* = 0.048) with small effect sizes (Improvement Rate Difference = 0.05–0.35). The intervention was found to be acceptable and showed moderate usability. Participants indicated they experienced improvements in reflection on feelings and changes in behavior, such as pausing and evaluating the situation.

**Conclusion:**

These preliminary results show that biocueing could be a promising addition to digital treatment platforms and help young people become more emotionally aware. Improvements should be made regarding the usability and acceptability of the smartwatch, as well as more extensive integration of the biocueing intervention with a digital treatment platform. It would be relevant to gain a better understanding of which individuals would benefit most from an additional biocueing intervention.

## Introduction

Approximately three-quarters of all mental health problems start before the age of 25 (1–3), and half of all episodes of mental health disorders start at the age of 14 (4, 5). Mental disorders make up for the number one burden of disease in young people (6). Untreated mental health disorders could lead to chronicity, comorbidity, lost potential, a lower social and occupational health, increased risk of suicide, and a lower quality of life for the individuals concerned (7, 8). On a broader scale, mental health disorders are associated with considerable economic consequences (9). Mental health costs are the single highest source of global economic burden in the world (10) and the Netherlands is estimated to spend around 5.7 billion euro annually on curative mental healthcare (11). Therefore, it is very important that early prevention and appropriate interventions are designed for young people (12) at the time when they are developing strategies to cope with stress and negative emotions (13). A very successful Australian initiative has implemented a comprehensive, evidence based youth focused indicative prevention program, which combines care in a stepped-care manner and blends peer-to-peer support with online counseling and therapy (Moderated Online Social Therapy (MOST+); (14–17).

Despite the success of MOST+ it is unknown whether this initiative can be translated to different countries and cultures. Currently, a study to implement the MOST+ platform in the Netherlands is being carried out under the name ENgage YOung people earlY (ENYOY) (18). In addition, most activities in the MOST+ platform require participants to have the ability to note fluctuating levels of stress and physiological arousal. Unfortunately, the ability to sense and feel stress and physiological changes (i.e., interoceptive sensitivity), might be especially impaired in the vulnerable adolescent target group (19–21). Therefore, additional tools that help young people note when physiological arousal is high and help them to apply methods to deal with this (such as behavioral experiments, mindfulness or muscle relaxation) would offer a potential further benefit to a wider group of adolescents. For this purpose biocueing might be an interesting addition. Biocueing is a non-invasive method to transform physiological parameters with a small electronic device into a visible, audible, or tactile signal, cued to the wearer (22). It automatically engages the user by prompts in daily stressful situations (23). The aim of the present study is to exploratory investigate the effects of biocueing as an addition to the moderated online social therapy-platform ENYOY for young people with emerging mental health problems.

During adolescence, the transitional period between child- and adulthood, young people go through a myriad of physical, social, and psychological changes (24–26). Brain areas involved in arousal and risk-reward perception rapidly develop, resulting in heightened stress responsivity and emotional reactivity (27). Fortunately, strategies to cope with stress and negative emotions also begin to form (28), with emotional awareness, (the ability to identify and label internal emotional experiences (29, 30), being an important building block in guiding individuals toward adaptive emotion regulation (19–21). Nonetheless, the functional change in arousal and risk-reward perception seems to precede the development of functional coping strategies, which creates a situation where, as well put by Steinberg (27), ‘an engine is started without yet having a skilled driver behind the wheel'. This discrepancy renders adolescents more vulnerable for negative emotions and psychological stress, and consequently heightens the risk of developing mental health problems (13). Indeed, research shows that there is a relation between stress and mental disorders (31). Less stress reactivity has been shown to predict symptom improvement in, for example, children with anxiety disorders (32) and adolescents with depressive symptoms (33). Moreover, different patterns in parasympathetic and sympathetic nervous system activation have been found to respond to different kinds of treatments (34).

It is of importance to take into account the multiple biological processes that make young people more vulnerable for psychological stress and further optimize treatment accordingly. In an attempt to reduce mental health problems among young people and reach them for indicative prevention mental health treatment, an Australian initiative has been successfully implemented (12). In more than 100 Headspace centers young people can find help for general health and education problems, drug use and (emerging) mental health complaints. Evidence-based interventions are provided in a stepped- care manner (17).

In addition to face-to-face interventions, Moderated Online Social Therapy (MOST+) has been developed to provide young people with comprehensive, adaptive, and integrated digital support. MOST+ merges peer-to-peer social networking; theory-driven and evidence-informed therapeutic interventions; expert clinician and vocational support; and peer support and moderation (14, 17, 35, 36). Findings from several pilot studies with young people diagnosed with (ultra-high risk for) psychosis, depression, social anxiety, and suicidal ideation have consistently shown high levels of feasibility, acceptability, engagement, and safety; and indicated effectiveness in a wide range of clinical and social outcomes [for a comprehensive overview, see: (14, 16, 17, 35)]. Additionally, MOST+ was found to be effective in improving vocational recovery and reducing utilization of emergency services in young people with a first episode of psychosis (16). Currently, a study to implement the MOST+ platform in the Netherlands is being carried out under the name ENgage YOung people earlY (ENYOY) (18). The MOST+ platform has been translated and adapted in cooperation with Dutch experts to fit the needs of young Dutch people, and will be continuously adapted to the wants and needs of participants over the course of the study.

With the goal of all young people benefitting fully from the platform, it would be of interest to look for adaptations or additions to the platform in the Netherlands. Although high levels of feasibility and acceptability were found for the use of the platform in Australia, not all users concluded that MOST+ was relevant to their needs (17), and engagement rates with the platform in young people with psychosis were found to be good, but not for all (80% engaged for the first 3 months, and 47% at 9 months) (16).

A factor that could play a role is the largely verbal content on the platform - though the platform also provides visual and audible exercises such as audio tracks and comics -, exercises are explained and information is given in text and metaphors, and the community is largely made up of verbal posts. However, not all individuals are equally verbally strong (e.g., (37, 38), and thus might not be able to understand its contents. Moreover, some of the exercises of the evidence-based therapies offered on the platform rely on interoception. For example, noting one's own thoughts and emotions (39) is required in the “reflective actions” on the platform. Possibly, some young people may not benefit because they have problems with interoception or, for some, interoceptive sensitivity might not have been fully developed yet (40). Another possible explanation for lower engagement rates could be that the intervention requires a certain amount of self-reliance of the individual, although monitored by a clinical moderator, namely to actively go online themselves. This might be challenging for some, for example for individuals with attention-deficit/hyperactivity disorder who have problems with, for instance planning [e.g., (41)], or individuals with relatively mild mental health complaints (42). Lastly, adherence rates for online interventions are found to be highly varying [e.g., 27.9 and 98% in (43)], for which a medium that could send reminders might contribute (44).

For individuals who are verbally less strong, have problems with interoception, and/or have problems with adherence, an intervention that addresses these individual factors may be helpful. Biocueing could be a suitable extra tool to support the development of coping strategies, as well as interoception, thereby strengthening an individual in stress- and emotion regulation (23).

Biocueing is a non-invasive method to transform physiological parameters, e.g., heartbeat or skin conductance, with a small electronic device - such as a smartwatch - into a visible, audible, or tactile signal, cued to the wearer (22). It automatically engages the user by prompts in everyday life stressful situations. These wearable devices can be helpful in supporting antecedent emotional regulation strategies in response to emotion evoking events and have been found to decrease self-reported stress-levels (23). De Looff and colleagues (45) found that in the 20 minutes leading up to an incident of aggression in individuals with borderline personality disorder, skin conductance level and the number of nonspecific skin conductance responses per minute rose significantly. This means that it is possible to detect certain interoceptive signals, thereby heightening emotional awareness and supporting reappraisal of emotions (20, 46), a process which is essential to mental health (46).

The aim of this study is to exploratory investigate the effects of biocueing as an addition to the moderated online social therapy-platform ENYOY for young people with emerging mental health problems. The objectives are three-fold: (1) to qualitatively explore whether biocueing could be of additional value to ENYOY; (2) to assess the effect of biocueing in combination with ENYOY on perceived stress and emotional awareness; (3) to evaluate the attitude of users concerning the usability, acceptance, and user-friendliness of the biocueing application and smartwatch in combination with the ENYOY-platform as an intervention.

## Methods

### Study Context

The present study took place within the context of the ENYOY-project [ENgage YOung people earlY; for a comprehensive summary, see (18)], where young people between the ages of 16 and 25 with emerging mental health complaints use the Dutch Moderated Online Social Therapy (MOST+) platform for 6 months. The goal of ENYOY is to offer young people a self-efficacious way of reducing their mental health complaints with digital interactive psychological interventions focused on using and developing personal strengths in combination with online counseling meetings with a psychologist and/or peer worker (a young person with lived experience with mental health complaints).

The ENYOY-project has received ethical approval from the Medical Ethics Review Committee (MERC) at Amsterdam University Medical Centers (AMC), the Netherlands (reference: NL66345.018.18), and was registered in the Netherlands Trial Register (ID NL8966). Written informed consent was obtained from all participants before inclusion to the study.

### Study Design

**Figure 1:**
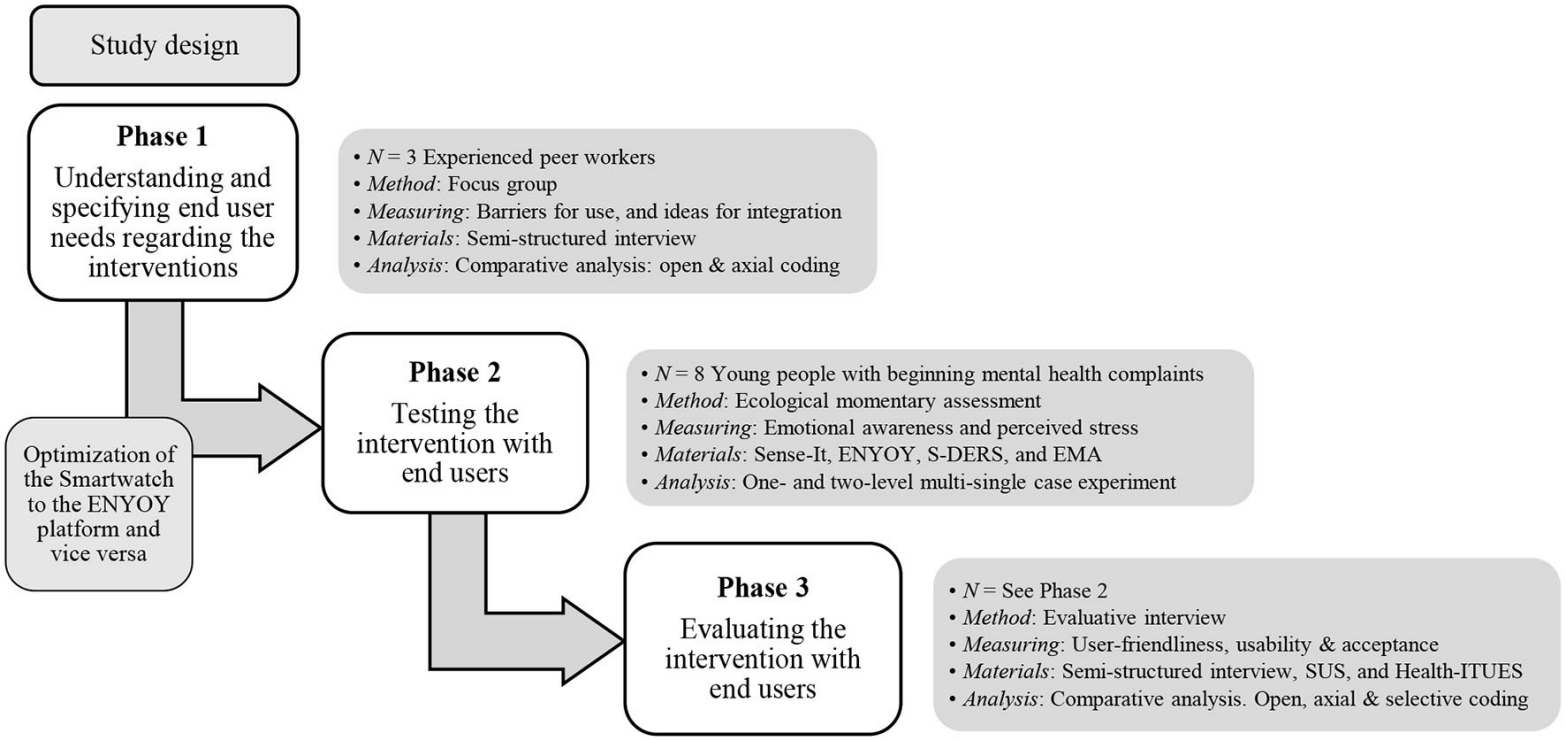
Study design. N, amount and type of participants; ENYOY, ENgage YOung people earlY project; S-DERS, State Difficulties in Emotion Regulation Scale; EMA, ecological momentary assessment; SUS, System Usability Scale; Health-ITUES, Health Information Technology Usability Evaluation Scale.

A user-centered (47) mixed method design was used consisting of three phases, see Figure 1. In the first phase (Phase 1–*Understanding and specifying end user needs regarding the interventions*), a qualitative design using a focus group, consisting of peer workers of the ENYOY-platform, was administered in which the requirements of end users in relation to the interventions were mapped and used to optimize the usage of the ENYOY-platform with the device and app used for biocueing (see materials > Sense-IT). In the experimental period (Phase 2–*Testing the intervention with end users)*, emotional awareness and perceived stress-levels were measured in real time using ecological momentary assessment (EMA). By sampling stress and emotional awareness in real time, EMA aims to minimize recall bias and maximize ecological validity (48). After the experimental period (Phase 3–*Evaluating the intervention with end users)*, user friendliness, usability, and acceptance of the interventions using a qualitative design. The Statement on Reporting of Evaluation Studies in health informatics framework will be followed in reporting this study to create a better understanding of the study flow with the different phases (49).

### Phase 1–Understanding and Specifying End User Needs Regarding the Interventions

#### Participants

Three peer workers of the ENYOY-platform with lived experience with similar mental health complaints as the end users participated. Being involved with the ENYOY-platform for over 4 years, the peer workers are experts in using the platform. They have been moderating the community of ENYOY and are in contact with participants since launch. The members of the focus group were aged between 22 and 29 years (M = 25.6, SD = 3.3), two were female and one male, and were all native Dutch speaking.

#### Materials

A semi-structured interview was conducted with questions regarding barriers for use, ideas for exercises on the platform, and ideas for integration of the biocueing intervention (see materials > Sense-IT) with the ENYOY-platform.

#### Procedure

Three out of four peer workers of the ENYOY-platform were able to participate in the focus group. In a focus group the informative source is a group of individuals and the heuristic value of this technique lies in the kind of interaction that emerges during the debate they have (50). End user needs were analyzed following the user-centered design framework (47), aiming to increase user acceptance and satisfaction, and decrease user errors and drop-out. The central question of the focus group was: What is the optimal way to combine the usage of biocueing via the Sense-IT/smartwatch with the ENYOY-platform? A researcher with experience and knowledge of the ENYOY-platform and the biocueing intervention led the focus group using open-ended questions such as: ‘How do you think the ENYOY-platform should be changed or adjusted to work well with a smartwatch?”, which was followed by a discussion in the focus group. Participants were invited to build on each other's answers. Afterwards, participants were sent a summary of their answers and were given the opportunity to make changes. The intervention used in Phase 2 was adjusted based on the feedback received from the peer workers (see Results, *Phase 1*).

#### Data Analysis

The qualitative data was analyzed using deductive thematic analysis (51). Predetermined themes, as assessed in the semi-structured interview, were formed into categories. Data that did not fit these predetermined categories were further coded openly using inductive coding (51).

### Phase 2–Testing the Intervention With End Users

#### Participants

A total of eight young people from the population of ENYOY users participated. The sample size was chosen with care, as using the biocueing intervention could be burdensome for some individuals when used for extended periods of time, and since the intervention has not yet been tested in combination with a treatment platform. Therefore, a relatively small sample size was used for this feasibility study to not unnecessarily burden participants. EMA was used with multiple measures per day per individual in order to maintain sufficient power, based on previous biocueing research (52). No additional inclusion criteria were administered (18). The exclusion criterion was the inability to wear the smartwatch during daytime (e.g., because of a job in healthcare or the catering industry). All participants were female, with a mean age of 22.38 years (*SD* = 2.13), and all had a formal education, split evenly between higher vocational education and university. Mental health problems were categorized by the clinical staging model (see (53) for the clinical staging model, and (18) for the operationalization in the ENYOY-study). Participants were included if the mental health complaints were found to be stage 1a (help-seeking individuals with mild symptoms and mild functional impairment) or 1b (people with attenuated syndromes with partial specificity, often with mixed or ambiguous symptoms and moderate functional impairment), and their occurrence was evenly divided over both categories. The time that a participant was on the platform ranged from 35 to 175 days [M(*SD)* = 112.38(52.03)].

#### Materials

##### Intervention

*Sense-IT*. The Sense-IT application (54) is designed to help an individual become aware of physiological parameters, such as heartbeat, as measured by the smartwatch. It notifies the user of changes in heartbeat by giving notifications and vibrations through a smartwatch (TicWatch Pro 3 GPS) and smartphone (Moto G30). The app was tailored to the needs of the user by changing the frequency and the message of the notification. The smartwatch ran on Android OS 11 and was connected to the smartphone by Bluetooth. The data on the smartphone was saved offline for privacy concerns. Through the smartphone, the EMA items were administered (see *questionnaires*).

*ENYOY*. Participants continued to have access to the ENYOY-platform. Young adults could use the plethora of options the platform has to offer: (1) therapeutic exercises based on positive psychology, acceptance and commitment therapy, and cognitive behavioral therapy; (2) biweekly online contact with a clinical moderator and/or peer worker that coaches the young adult to work on their mental health problems; (3) a community newsfeed with peers and peer workers.

##### Questionnaires

*Emotional Awareness*. To measure emotional awareness, the Dutch version of the State Difficulties in Emotion Regulation Scale [S-DERS, (55)] was used. This 21-item questionnaire, scored on a 5-point Likert scale (1 = “Not at all” to 5 = “Completely”), was adjusted to EMA for this study, meaning the questions were adapted for answering on a smartphone. The Awareness and Clarity subscales were used: These scales relate to emotional awareness and the other constructs of the questionnaire are beyond the scope of this study. Examples of these subscales, respectively, are: “I am paying attention to how I feel” (α = 0.79) and “I am confused about how I feel” (α = 0.65). Subscale scores were calculated by summing the scores and averaging: A high score on the aggregated subscale indicates high awareness of emotion.

*Perceived Stress*. To measure perceived stress, the Dutch EMA survey item “I feel stressed” was used, scored on a 0 to 5-point scale (“Not at all” to “Very much”). This item measures negative affect with high arousal and was extracted from the EMA repository of Kirtley and colleagues (56). A higher score on this scale indicates higher perceived stress-levels.

#### Procedure

All participants of the ENYOY-study that were online at the time of this study were asked if they were interested in wearing a smartwatch to cope with their stress-levels in addition to their participation on the ENYOY-platform. Out of 40 participants, eight had the time and interest to participate. A total of four appointments with the research assistant took place (a set-up meeting, two calls to check whether the Sense-IT was working properly, and a meeting to evaluate the usability of the intervention). Participants were sent a package by mail containing the hard- and preinstalled software.

During the set-up meeting, participants were asked to choose two moments every day, one in the morning and one in the evening, to prompt the EMA questions. The baseline heart rate of the participant was determined using the protocol used by ter Harmsel and colleagues (57) and participants were asked what they would do when experiencing an above average heartbeat. The ENYOY-platform was mentioned by the researcher to cope with a high heart rate. Specific interventions of the platform that could help reduce stress according to the participant were added to the “toolkit,” so that exercises could be accessed easily. The condition alternated over the course of 20 days, in the first 10 days for all participants “biofeedback off” (control), and in the last 10 days “biofeedback on” (experimental). Participants were prompted twice daily to answer the EMA items regarding perceived stress-levels and emotional awareness during both conditions.

#### Data Analysis

Data from the subscales Emotional Awareness and Emotional Clarity were aggregated per participant, after which a multi-single case experimental design (multiSCED) was used for measuring perceived stress-levels and emotional awareness per participant (one-level) and overall (two-level). This statistical test is produced using the MultiSCED-tool by Declercq et al. (58). For each participant, condition and time were used to predict stress and emotional awareness by means of the following regression formula:


(1) $$\begin{array}{l} \begin{array}{c} Y_{i}\;=\;\beta 0\;\left(Intercept_{i}\right)\;+\;\beta 1\;Time{\;}_{i}+\;\beta 2\;Condition{\;}_{i}\\ +\;\beta 3\;\left({Time\;\times \;Condition}_{i}\right)\;+\;e_{i} \end{array} \end{array}$$

The subscript *i* denotes the measurement nested within a case. To determine the overall effect of bio cueing, condition and time were used to predict stress and emotional awareness by means of the following regression formula,


(2) $$\begin{array}{l} \begin{array}{c} Y_{ij}\;=\;\beta 0\;\left(Intercept_{i}\right)\;+\;\beta 1\;{Time\;}_{ij}+\;\beta 2\;Condition{\;}_{ij}\\ +\;\beta 3\;\left({Time\;\times \;Condition}_{ij}\right)\;+\;e_{ij}\backslash n \end{array} \end{array}$$

The Improvement Rate Difference (IRD; (59) was used to calculate the effect sizes. The IRD is defined as the improvement rate of the treatment phase minus the improvement rate of the control phase.

### Phase 3–Evaluating the Intervention With End Users

#### Participants

See Phase 2.

#### Materials

##### Evaluative Interview

*User-Friendliness*. To measure user-friendliness, semi-structured interviews took place in which questions were asked regarding the participants' experience with the hard- and software. These questions were based on earlier research by Derks and colleagues (52).

*Usability and Acceptance*. To measure the usability and acceptance of the Sense-IT, two questionnaires were administered. The Dutch version of the System Usability Scale [SUS; (60)] is a 10-item questionnaire scored on a 5-point Likert scale (1 = “Strongly disagree” to 5 = “Strongly agree”), measuring the general usability and acceptance of an intervention. An example of an item is “I think that I would need the support of a technical person to be able to use this system.” The composite measure ranges between 0 and 100, with higher scores indicating higher usability. Scores below 50 indicate non-acceptance, while scores above 50 indicate acceptance (61).

The Health Information Technology Usability Evaluation Scale [Health-ITUES; (62)] is a 20-item questionnaire, scored on a 5-point Likert scale (1 = “Strongly disagree” to 5 = “Strongly disagree”), which focuses on usability of mobile health technology, and has four subscales: Impact, Perceived Usefulness, Perceived Ease of Use, and User Control. An example item of Impact is: “I think ENYOY combined with the Sense-IT would be a positive addition for persons living with beginning mental health problems” (α = 0.85). A high score on User Control and Perceived Ease of Use captures the user-system interaction, whereas Perceived Usefulness evaluates task accomplishments through system use. Impact refers to the system's impact on daily life. A high overall score indicates a higher perceived usability of the technology.

#### Procedure

After the 20 day experimental period, a meeting took place between the research assistant and the participants of phase 2 to evaluate the intervention by means of the semi-structured interview, the SUS, and Health-ITUES. The interviews were conducted through video call in a secured Microsoft Teams environment by a research assistant with experience and knowledge of the ENYOY-platform and the biocueing intervention.

#### Data Analysis

The qualitative data was analyzed using deductive thematic analysis (51). Predetermined themes, as assessed in the semi-structured interviews, were formed into categories. Data that did not fit these predetermined categories were further coded openly using inductive coding (51). Finally, selective coding was used to exploratory determine possible relations and connections between the data and categories (63). Considering the SUS and Health-ITUES averages and ranges were calculated and interpreted.

## Results

### Phase 1–Understanding and Specifying End User Needs Regarding the Interventions

The following adaptations were implemented as a result of the focus group for the optimization of the smartwatch to the ENYOY-platform and vice versa. For a full overview of the results of the thematic analyses, see Supplementary Appendix A.

Firstly, in the manual of the Sense-IT the benefits of the intervention were specified, as well as steps to personalize the smartwatch to the issues the young person struggles with. Secondly, access to the ENYOY-platform was made easier by (a) making exercises available offline, (b) helping young people fill their ENYOY toolkit with useful exercises beforehand, and (c) a link was added from the Sense-IT to the ENYOY toolkit. Third, ENYOY was adjusted to the smartwatch and vice versa to help young people slow down and reflect by (i) creating an exercise that helps differentiate between emotions, (ii) questions were prompted about young people's needs and experienced emotions on the smartwatch, and (iii) it was individually addressed whether or not the participant also wanted to be signaled when their heartbeat is low. Finally, in order to deal with obstacles for use, the following steps were taken: (1) young people were helped with deciding whether a smartwatch fits their lifestyle and were helped creating a new habit by linking the intervention to old habits during the introductory meeting; (2) it was addressed in the introductory meeting that hypervigilance could increase stress and that there is no correct or false way of experiencing stress, and (3) participants were contacted 2 days into the experiment to ask about the functioning of the smartwatch and Sense-IT application.

### Phase 2–Testing the Intervention With End Users

Compliance, as defined by the ratio of the number of measurement occasions that participants completed in relation to the maximum number (64), was on average 78.8%, ranging from 59.4% to 100%. An exact McNemar's chi square test determined that there was a significant difference in compliance between the control condition (86.1%) and the experimental condition (70.8%), *p* < *0.0*01. In order to improve the quality and reliability, missing data (*N* = 64) was imputed using the Expectation-Maximization (EM) method, following Chen, Feng, Wu, and Peng (65).

The assumption of normality was tested by depicting a histogram of standardized residuals, which indicated that the data contained approximately normally distributed errors, as did the normal probability plot (P-P plot of) standardized residuals, which showed data points followed a straight line.

**Figure 2:**
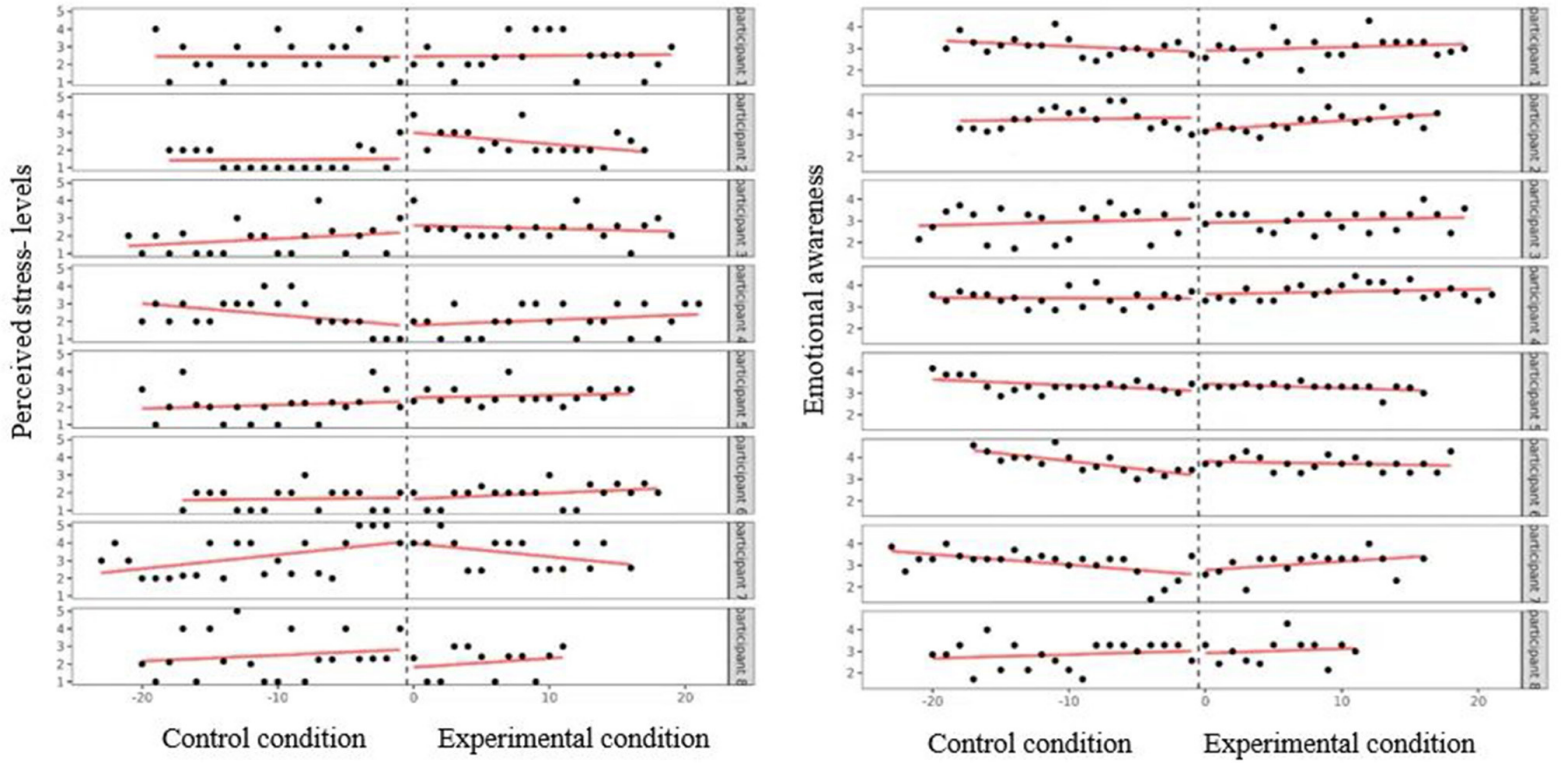
Perceived stress-levels (left) and emotional awareness (right) as a function of condition and measuring time per participant.

Table 1 displays an overview of the results from the level-one ordinary least squares regression analysis. Regarding perceived stress-levels, in the control condition (biocueing off) no significant effect was found for six out of eight participants (see Figure 2; participant (P) 1: *B* = −0.001, *SD* = 0.04, *p* = 0.980; P 2: *B* = 0.005, *SD* = 0.03, *p* = 0.872; P 3: *B* = 0.038, *SD* = 0.03, *p* = 0.178; P 5: *B* = 0.021, *SD* = 0.03, *p* = 0.480; P 6: *B* = 0.007, *SD* = 0.03, *p* = 0.802; *P* 8: *B* = 0.034, *SD* = 0.04, *p* = 0.450). One participant showed an increase (P 7: *B* = 0.079, *SD* = 0.03, *p* = 0.009) and one a decrease (P 4: *B* = −0.065, *SD* = 0.03, *p* = 0.047). In the experimental condition (biocueing on), no significant effect of the intervention on stress-levels was found for six out of eight participants (P 1: *B* = 0.007, *SD* = 0.06, p = 0.903; P 2: *B* = −0.068, *SD* = 0.04, *p* = 0.129; P 3: *B* = −0.018, *SD* = 0.04, *p* = 0.177; P 5: *B* = 0.008, *SD* = 0.05, *p* = 0.868; P 6: *B* = 0.025, *SD* = 0.04, *p* = 0.511; P 8: *B* = 0.017, *SD* = 0.11, *p* = 0.870). One participant showed an increase (P 4: *B* = 0.094, *SD* = 0.04, *p* = 0.03) and one participant showed a decrease (P 7: *B* = −0.154, *SD* = 0.06, *p* = 0.01). Improvement rate difference (IRD) effect sizes ranged from 0.16 to 0.35, which is indicative of a small effect size of the combination of biocueing and ENYOY on perceived stress-levels. Results from the level-two ordinary least squares regression analysis show that, across cases, there was no significant effect of the intervention on perceived stress-levels, (*B* = −0.020*, SD* = 0.03*, t*(6.39*)* = –*0.6*1*, p* = *0.5*62). These findings indicate that biocueing in combination with ENYOY does not decrease perceived stress-levels among participants.

**Table 1:** Summary of study results for dependent variables per participant and per condition.

		**Outcome**			
		**Perceived stress levels**		**Emotional awareness**	
**Participant**		**Control condition**	**Experimental condition**	**Control condition**	**Experimental condition**
1	N (measuring points)	19	20	19	20
	Intercept B (SD)	2.426 (0.47)	2.440 (0.63)	2.831 (0.22)	2.901 (0.30)
	Slope B (SD)	−0.001 (0.04)	0.007 (0.06)	0.027 (0.02)	0.044 (0.03)
	IRD	–	0.18	–	0.18
2	N (measuring points)	18	18	18	18
	Intercept B (SD)	1.507 (0.34)	2.976^**^ (0.57)	3.799 (0.21)	3.212^*^(0.28)
	Slope B (SD)	0.005 (0.03)	−0.068 (0.04)	0.009 (0.02)	−0.034 (0.03)
	IRD	–	0.56	–	0.17
3	N (measuring points)	21	20	21	20
	Intercept B (SD)	2.212 (0.35)	2.581 (0.48)	3.099 (0.28)	2.902 (0.39)
	Slope B (SD)	0.038 (0.03)	−0.018 (0.04)	0.016 (0.02)	0.002 (0.03)
	IRD	–	0.46	–	0.27
4	N (measuring points)	20	22	20	22
	Intercept B (SD)	1.721 (0.38)	1.783 (0.51)	3.380 (0.17)	3.378 (0.23)
	Slope B (SD)	–0.065^*^(0.03)	0.094^*^(0.04)	−0.002 (0.01)	0.013 (0.02)
	IRD	–	0.05	–	0.28
5	N (measuring points)	20	17	20	17
	Intercept B (SD)	2.323 (0.35)	2.540 (0.49)	3.090 (0.12)	3.413 (0.17)
	Slope B (SD)	0.021 (0.03)	0.008 (0.05)	–0.027^*^(0.01)	0.008 (0.02)
	IRD	–	0.67	–	0.35
6	N (measuring points)	17	19	17	19
	Intercept B (SD)	1.713 (0.30)	1.645 (0.40)	3.135 (0.17)	3.818^**^ (0.42)
	Slope B (SD)	0.007 (0.03)	0.025 (0.04)	–0.071^***^(0.02)	0.061^**^(0.02)
	IRD	–	0.22	–	0.16
7	N (measuring points)	23	16	23	16
	Intercept B (SD)	4.127 (0.39)	3.975 (0.58)	2.537 (0.22)	2.776 (0.32)
	Slope B (SD)	0.079^**^(0.03)	–0.154^*^(0.06)	–0.049^*^(0.02)	0.039^**^(0.03)
	IRD	–	0.31	–	0.31
8	N (measuring points)	20	12	20	12
	Intercept B (SD)	2.839 (0.53)	1.810 (0.82)	3.033 (0.29)	2.917 (0.44)
	Slope B (SD)	0.034 (0.04)	0.017 (0.11)	0.018 (0.02)	0.020 (0.06)
	IRD	–	0.33	–	0.53

*N, amount of; M, mean; SD, standard deviation. Both intercept and slope refer to respectively the unstandardized B**−**coefficient and the change in the unstandardized B**−**coefficient per time unit*.

* *p ≤ 0.05*,

** *p ≤ 0.01*,

*** *p ≤ 0.001. IRD, Improvement Rate Difference. Numbers in bold highlight the significant findings*.

No significant effect for emotional awareness in the control condition was found for five out of eight participants, see Table 1 and Figure 2 (P 1: *B* = −0.027, *SD* = 0.02, *p* = 0.17; P 2: *B* = 0.009, *SD* = 0.02, *p* = 0.644; P 3: *B* = 0.016, *SD* = 0.02, *p* = 0.487; P 4: *B* = −0.002, *SD* = 0.01*, p* = *0.8*91; P 8: *B* = *0.0*18, *SD* = 0.02, *p* = *0.4*56). Three participants showed a decrease in levels of emotional awareness (P 5: *B* = −0.027, *SD* = 0.01, *p* = 0.012; P 6: *B* = −0.071, *SD* = 0.02, *p* < 0.001; P 7, *B* = −0.049, *SD* = 0.02, *p* = 0.004). In the experimental condition, no significant effect was found for six out of eight participants (P 1: *B* = 0.044, *SD* = 0.03, *p* = 0.111; P 2: *B* = 0.034, *SD* = 0.03, *p* = 0.211; P 3: *B* = 0.002, *SD* = 0.03, *p* = 0.941; P 4: *B* = 0.013, *SD* = 0.02, *p* = 0.484; P 5: *B* = 0.008, *SD* = 0.02, *p* = 0.612; P 8: *B* = 0.002, *SD* = 0.06, *p* = 0.966). Two participants showed an increase in levels of emotional awareness (P 6: *B* = 0.061, *SD* = 0.02, *p* = 0.008; P 7: *B* = 0.039, *SD* = 0.03, *p* = 0.007). The IRD for significant results ranged from 0.05 to 0.31, which is indicative of a small effect size. Results from the level-two ordinary least squares regression analysis showed that, across cases, there was a significant fixed interaction effect of time and condition on emotional awareness (*B* = 0.030, *SD* = 0.01, *t* (6.71) = 2.42, *p* = 0.048), indicating that in the experimental condition, emotional awareness increased significantly over time. These findings indicate that the combination of ENYOY and biocueing could increase emotional awareness.

### Phase 3–Evaluating the Intervention With End Users

Thematic analysis from the evaluative interviews yielded the following themes: usability of the Sense-IT, integration of the Sense-IT and the ENYOY-platform, and ideas for improvement of the Sense-IT and the ENYOY-platform.

The overall usability of the Sense-IT could be rated as moderate with reported usability issues. On the one hand, positive experiences were reported. All participants mentioned that they reflected more on how they were feeling and three noted that this helped them understand their emotions better. All participants mentioned making an effort to change something in their behavior because of the biofeedback, such as pausing and evaluating the situation or doing a breathing exercise. Scores on the Health-ITUES indicated that participants judged the impact and usefulness of the intervention to be moderate (*M* = 3.93, *SD* = 0.43; *M* = 3.71, SD = 0.42, see Table 2). These scores indicate that the intervention impacted the participant's daily life moderately and participants were moderately able to accomplish tasks through system use.

**Table 2:** Descriptive statistics for subscales of the Health ITUES subscales.

**Scale**	**M (SD)**	**Median (range)**
**Overall Health-ITUES score**	3.69 (0.30)	3.75 (3.25–4.20)
HI score impact	3.93 (0.43)	3.66 (3.33–4.67)
HI score perceived usefulness	3.71 (0.42)	3.56 (3.11–4.33)
HI score ease of use	3.63 (0.46)	3.80 (2.80–4.20)
HI score user control	3.29 (0.74)	3.00 (2.00–4.33)

*HI, Health-ITUES; M, mean; SD, standard deviation*.

P5: “*I liked using it. For me personally, the watch was quite big, as I have small wrists. I liked the notifications, like: “Hey, watch out, your heartbeat is quite high, is something the matter?” [...] having the device tell me that something might be happening [within me] helped me to be mindful of that. I liked that very much.”* ^1^

On the other hand, participants mentioned several technical problems. Most participants [5] noted difficulties with the interface, resulting from problems with making notes in the app [2], not receiving text notifications [1], the watch display changing to default settings [1], and difficulties in figuring out how to know when the biocueing intervention was turned on [1]. Furthermore, most participants [5] expressed doubts about the accuracy of the heartbeat measurements, with participants receiving too much [4] or too little biofeedback [1], and three participants mentioned that their baseline measurement failed multiple times.

P6: “*Sometimes I got a notification while I was sitting still, it said that my heartbeat was too high and I thought ‘That's weird', because I wasn't doing anything stressful. There was also a situation in which I was stressed but I didn't see that on the watch”*

Lastly, half of the participants mentioned that the usage of the intervention was disrupted due to limited battery life of the smartwatch. Half found the appearance of the smartwatch acceptable, others noted that the smartwatch was too big [2], did not fit their style [1], or found the strap uncomfortable [1]. The average system usability score (SUS) of the group was above the cut-off score (*M* = 63.78, *SD* = 10.96), meaning the intervention was permissible in terms of acceptance, though indicating that there are usability issues that are cause for concern. Results from the Health-ITUES yielded a moderate usability score (*M* = 3.69, *SD* = 0.30, ranging from 2 to 4.33; also see Table 2 for average scores per subscale).

Regarding the integration of the Sense-IT and the ENYOY-platform, the breathing exercises from the ENYOY-platform seemed the most suitable choice when stressed and were done by most participants [7]. Half of the participants did not use the ENYOY-platform during the study, reasons being not having thought about it or not having time [2], finding it difficult to use ENYOY due to double verification which is used for logging in [1], or already having used the ENYOY-platform extensively in the past [1]. Scores on the Health-ITUES were reflective of this fact, as user control and ease of use was judged to be moderate (*M* = 3.63, *SD* = 0.46, see Table 2), indicating that the user-system interaction was not optimal.

P2: “*Well, I haven't thought about it [using the ENYOY-platform], and often when it [the watch] vibrated, I wasn't near my laptop. I like it better to use it on my laptop than to use it on my phone. I didn't open my laptop the past week, so that must've played a role.”*

Of the participants that had used the platform, most noted that they used the platform on their laptop [3] and that they planned time in their agenda to go on the platform [2]. The most used online tools were the toolkit [2] and the explore function [2]. Participants expressed their need for exercises that are short [2], are adapted to their needs [1], are practical [1], or include psycho-education [1]. Half of the participants mentioned that they did not always carry the research smartphone with them, which hindered them from using the intervention fully. They mentioned that they would use the Sense-IT more often if the research smartphone was directly connected to the ENYOY-platform or if they could use their own smartphone [both of which were not possible in this study because the research smartphone was an ‘offline' device for privacy concerns (see methods)].

P5: “*I used it [the ENYOY-platform] once or twice, because this phone wasn't connected to the internet, which made it more of an effort, because then I had to see when I receive a notification and ask myself “why, when?” and then I have to use the platform here [in the bedroom] on my laptop. So for me, it's a bit of an obstacle, because there are more actions involved.”*

Some participants thought they would be more inclined to use ENYOY if there were an app [3], if logging in was made easier [1], and if reminders for using the platform were given [1].

Participants gave many suggestions for improvement of the Sense-IT and the ENYOY-platform to further integrate the two interventions and to increase user experience (see Table 3). Important suggestions referred to the accessibility of the ENYOY-platform, the integration between the platform and the Sense-IT, and bugs in the Sense-IT.

**Table 3:** Overview of bugs, problems and proposed solutions from the semi-structured evaluative interviews.

**Intervention type**	**Problem**	**Proposed solution**
ENYOY	Not all participants used the platform during the study, because they found it difficult to log in	Make an app
		Make the web-app version of ENYOY more accessible (e.g., by incorporating an explanation of how to set the webpage on the home screen of a smartphone)
		Explain how to save double verification log in credentials/personalize double verification log in
	Not all participants used the platform during the study, because they had no time or forgot about it	Set reminders of suggested exercises
		Engage participants more by adding gamification elements to the platform to create positive feedback from the platform (e.g., Balance or Duo Lingo apps)
		Integrate the therapy path and explore function more (suggested exercises)
		Add quizzes to make the content more interactive and test whether or not young people understood the content
		Make sure translations on the platform are well executed
Sense-IT and smartwatch	The last heartbeat measurement was not intuitively visualized	It is less important to be able to click on the last measurement, as this often was not the most important measurement – it may be a better idea to show the last high or last low heartbeat measurement
	The baseline measurement was not adequately measured in many instances	-
	The movement sensor of the Sense-IT did not work properly	This problem is fixed once the intervention is used on a personal smartphone, as the app then is able to pinpoint the exact location through wireless internet connection
	The smartwatch had to be charged often (once daily)	-
	The Sense-IT at times switched off automatically	-
	The Sense IT watch display sometimes switched back to the default watch display	-
	Making notes further back than a few days was bugged	-
Combination ENYOY, Sense-IT, and smartwatch	Not all participants used the platform during the study because there were many actions involved to get to the platform	Provide a link from the Sense-IT to the ENYOY-platform on the smartphone
		Use the Sense-IT application on one's own smartphone
		Integrate a heartbeat measurements overview in the ENYOY dashboard

*“-” means that participants did not come up with a solution for this problem*.

## Discussion

The focus of this study was to investigate the usability, feasibility, and effects of biocueing in addition to the moderated online social therapy-platform ENYOY in young people with emerging mental health problems, following a user-centered (47) mixed-method design framework. The current study had three objectives: 1) to qualitatively explore whether biocueing could be of additional value to ENYOY; 2) to assess the exploratory effect of biocueing in combination with ENYOY on perceived stress and emotional awareness; 3) to evaluate the attitude of users concerning the usability, acceptance, and user-friendliness of biocueing in combination with ENYOY as an intervention.

The findings from the phase 1 (*Understanding and specifying end user needs regarding the interventions*) focus group indicated an additional value of a biocueing intervention to ENYOY and resulted in several suggestions regarding briefing, access, and integration for adaptation of the smartwatch to the ENYOY-platform and vice versa, which were implemented for phase 2 (*Testing the intervention with end users*). The compliance rate of phase 2 was on average 78.8%, which is similar to compliance rates in patient populations (66), and slightly higher then compliance rates for digital therapy platforms for youths [63,81% on average, (43)]. Exploratory findings of phase 2 showed different individual effects of biocueing on perceived stress and emotional awareness. Overall, no effects were found on perceived stress-levels, suggesting that the current combination of ENYOY and biocueing does not decrease perceived stress in young people. Positive exploratory effects were found on emotional awareness, suggesting that the combination of ENYOY and biocueing could increase emotional awareness and thereby possibly poses a relevant addition to a digital treatment platform. Nonetheless, some caution is advised because of the small sample size of this explorative feasibility study, and since Improvement Rate Difference [IRD; (59)] showed small effect sizes of the intervention.

Results from phase 3 (*Evaluating the intervention with end users*) showed improvements on reflection on feelings and positive changes in behavior - such as pausing and evaluating the situation, doing an exercise on ENYOY, and having a rest - following the biocueing intervention combined with the use of ENYOY. Participants preferred exercises on the ENYOY-platform that were short, adapted to their needs, practical, or included psycho-education. The most common tools used were breathing exercises, the toolkit (a library where the young person can save his/hers favorite exercises), and the explore function (a quick search function to show exercises per category, e.g., “rumination” or “stress”). Not all participants used the platform during the experimental phase, a reason being that the integration of the two interventions was very rudimentary due to the early implementation. These kinds of new intervention products are also known as Minimum Viable Products (MVP), which are first versions of a product or service which are being delivered to a target group as early as possible. The most common issues for MVPs are the non-optimal technical support and lack of resources (67), as was the case in this study. The intervention was found to be permissible in terms of acceptance and showed a moderate usability, with reported usability issues. The most common issues that were reported concerned the use of the smartwatch (e.g., technical issues, difficulties with the interface, doubts about the accuracy of the heart rate measurements, and limited battery life), and design of the smartwatch (appearance not fitting their style and lacking wearing comfort), as well as the non-optimal integration of the smartwatch and the ENYOY-platform (not being available on the same device). Main suggestions for improvement were related to the accessibility (e.g., the option to use both interventions on one device, within one app, and add reminders), engagement of users (e.g., adding gamification elements, create positive feedback for the platform, and make use of fun elements such as a quiz), and the integration (e.g., show heartbeat measurements of the smartwatch on the ENYOY dashboard).

As indicated by the results, the intervention significantly increased emotional awareness, and did not significantly decrease perceived stress. This means that the biocueing intervention in addition to ENYOY seems to make participants more aware of their (negative) emotional states and does not seem to play a part in decreasing perceived stress. The last is contrary to previous research where decreases in self-reported stress-levels were found (23). There are several factors that could play a role in this finding. First, stress was measured using one survey item, asking participants to indicate their stress-levels on Likert scale. It is questionable whether a single survey item, prompted twice a day, can accurately encapsulate a multifaceted concept like stress (68). Even though subjective measures have their obvious limitations, their merit lies in the ability to measure beliefs, thoughts, emotions, and attitudes about stress (69). Stress is not just the simple product of a perceived threat; according to the Transaction Model, stress is a product of the antecedent personal resources and external stressors, mediated by coping, followed by both short- and long-term effects (70). Studying the person-environment interaction and coping may provide additional valuable insight on the way these factors play a role in stress-reduction through biocueing. Furthermore, participants participating in the intervention had relatively low levels of stress, as indicated by the low levels of stress in the control condition. Therefore, stress had a low mean score and had little room for change. This little room for change was emphasized by the short period of time of the experiment: two weeks may not be enough to observably change stress in participants. Additionally, there were no fixed time frames for the measurements. This means that participants were free to answer the stress-related survey item any time after the question was prompted. Presumably, participants who experience heightened stress may not be able to direct their time toward answering the survey question and therefore answer the question later when heightened stress has subsided.

Even though emotional awareness plays a role in stress reduction, being more emotionally aware alone may not automatically cause someone to experience less stress without active attempts to reduce it (71). A biocueing wearable functions as an indicative system that provides the user with feedback on symptoms associated with stress, such as heightened heart rate through skin conductance. Participants that experience stress, but do not make an effort to reduce it through meaningful effort, may therefore become more aware of aversive feelings, but not experience relief from it. To emphasize, only half of all participants used ENYOY during the experimental phase, which raises the question how many participants made an effort to actively reduce stress if any at all. Participants felt that the integration of Sense-IT and ENYOY was lacking. Participants could not use their own phone and carried a research phone that was not connected to the internet or offering direct access to the ENYOY-platform for privacy concerns (an ‘online' device is more prone to data leaks and this was found too big a risk for the target population and the nature of their complaints), which was seen as a limiting factor in terms of usability. Participants mentioned they were more likely to use ENYOY if these issues were addressed and participants did not have to switch devices to access ENYOY. Furthermore, increased awareness of bodily sensations may adversely increase anxiety if they are interpreted catastrophically (72). Some young people might stop wearing their smart watches because the watches disturb their life by making them too 'aware' of their stress and situation. Finally, in the field of clinical psychology experiencing negative emotions is seen more and more as being a normal part of life. A stance more toward accepting these and learning how to deal with them might provide individuals with a more fulfilling life than striving to get rid of them [e.g., (73)]. Moreover, one of the keystones of ENYOY is normalizing difficult emotions (18). In this way, one could wonder whether the goal of biocueing and ENYOY should be to reduce stress, or to provide ways in how to deal with the stress that a person has in his or her life. For future research it would be of interest to not only measure stress-levels, but, more importantly, coping with stress.

The individual differences in effects of the intervention could have multiple reasons. First of all, in individual cases it is uncertain whether results are due to progress rather than time [(74); also see Limitations]. Moreover, individual differences in baseline stress-levels and emotional awareness [e.g., (75, 76)] could mean that for some there was less room for improvement (77) and for others that 10 days of biocueing was insufficient. Interestingly, three out of eight participants showed a decrease in emotional awareness in the control condition, of which two subsequently showed an increase in the experimental condition. This reinforces the idea that the intervention itself changed outcomes rather than time. Moreover, biocueing might not be a suitable intervention for all. If an individual already has a hypersensitivity toward one's internal state and/or experiences panic because of bodily sensation [e.g., for individuals who suffer from illness anxiety disorder, see (78); or individuals who are diagnosed with bipolar disorder, see (79)], biocueing could exacerbate this focus and panic even more. Another example regards individuals with problems with interoceptive sensibility (the capacity to focus on internal sensations and take them into consideration), such as individuals with autism and/or alexithymia, where difficulties are found in discriminating among interoceptive signals, which might result in difficulties understanding one's bodily states (80). For these individuals, a biocueing intervention could be especially challenging since it focuses mainly on interoceptive accuracy (a higher heartbeat is identified) and not on interoceptive sensibility (what does this mean to me?). Extra treatment regarding *how* to interpret, distinguish, and deal with these sensations and emotions would be required (80). Although ENYOY offers some exercises along these lines, this might be insufficient for some. All in all, the individual differences in effects indicate that the biocueing intervention might not be suitable for every individual, which should be addressed before providing individuals with a smartwatch.

A main point that might have countered the true added effects of biocueing to ENYOY is the usability of the biocueing intervention. The overall usability of the intervention was found to be moderate, with reported usability issues. Even though participants reported that the intervention helped them reflect more on how they were feeling or helped them in understanding their emotions, several issues were reported that impacted the usability of the smartwatch. Half of all participants found the smartwatch either too large, not fitting their style, or too uncomfortable. These factors, “aesthetics,” play an important part in deciding to either “use or lose” the smartwatch (44, 81, 82), even more so in adolescents who highly value the aesthetics of wearables (83). Therefore, it is important that the smartwatch fits a participant's sense of style to increase usability, which can either be achieved through customizable options or a choice of different styles. The Sense-IT application which was used in the study works on any Android OS watch, however for the purpose of this study all participants received the same smartwatch. One could imagine that a flashy self-chosen smartwatch linked seamlessly to a digital platform would significantly improve the link between signaling and outcomes.

### Limitations and Strengths

Several strengths and limitations of this study should be highlighted. A limitation of this study is that not all suggestions opted by the phase 1 focus group could be implemented for the intervention due to missing technical support and resources. This could have contributed to the lower usability and acceptance rates and lower use of ENYOY, and is a missing requirement that is necessary for a user-centered design (47). However, we hope that our findings could provide useful information for future research and implementation. Another limitation of this study are the compliance rates. Over 20% of measurements were missing and significantly more data was missing in the experimental condition. This was dealt with using Expectation-Maximization; a method that is found to keep the power sensitivity stable in case of missing data, even up to 40% (65). Additionally, an AB (A control phase, B experimental phase) approach was opted, which means it cannot be said with certainty that *individual* effects in phase B are due to progress rather than time. For this, at least three phase changes (ABAB) are necessary. This was compensated by using the multiple single-case design which eliminates the need to return to baseline (74), and enables to make conclusions about the intervention at group-level. Moreover, this study had a relatively small sample size. This was deemed fit for the type of research (an explorative feasibility study), and was chosen to not unnecessarily burden participants. Nonetheless, the small sample size could have affected the reliability of the results. The contrasting direction of the individual responses found in the level-one analysis of phase 2 of this study are also indicative of a greater sampling variability. Lastly, all participants were female and had followed higher vocational education or university. This could have influenced the representativeness of the user group in relation to the overall population.

A strength of this study is its user-centered mixed-method design. The end users played a central role in every iteration of the study and were involved in the developmental process of the integration of the ENYOY-platform and the smartwatch (co-creation). This provided a deeper understanding of psychological, social, and ergonomic factors related to the used technology. Though a pitfall of this could be that the end product would be too specific for general use and less transferable to other clients. Moreover, this method has been found to improve effectiveness, efficiency, and safety of technological products (47). The use of mixed-methods also provided us with rich, comprehensive data by integrating qualitative and quantitative data (84). The use of ecological momentary assessment further allowed us to measure in real-time, which minimizes recall bias and maximizes ecological validity (48).

Considerable efforts were made to increase and ensure quality control. The Sense-IT app has already been used in previous studies after being developed with well-recognized research methods (52, 57) which decreases the risk of statistical anomalies due to hard- or software factors. In addition, the Sense-IT was built in accordance with the Medical Devices Directive (MDD) and Active Implantable Medical Devices Directive (AIMDD) ensuring that Sense-IT has to meet certain quality standards (54). Additionally, the SUS (60) and S-DERS (55) have shown adequate validity and reliability making it useful tools for this study. Furthermore, the Health-ITUES (62) which had no original Dutch version, was carefully translated back-and-forth (English-Dutch, Dutch-English, and English-Dutch) between four independent translators to limit diminished validity and reliability through mistakes in translation.

### Future Research

For future research, foremost, usability studies are recommended to improve the smartwatch and integration with a digital platform. This study highlighted the need for research with the goal of full optimization, preferably in co-creation with end-users [see for example (52, 85, 86)]. It would be of further relevance to gain a better understanding of which individuals would, and which would not, benefit from a biocueing intervention (in addition to a digital treatment platform). In the future, it would be valuable to connect the ENYOY-platform to the smartwatch in such a manner that the activities of young people on the platform could be followed to see which part(s) of the platform is/are most effective to relieve stress. Moreover, multidimensional assessments of both stress and emotional awareness could provide valuable new insights [also see (69)]. It would also be of interest to investigate whether an optimized detection of stress and corresponding interventions lead to greater effects; and to evaluate via rigorous designs whether biocueing improves outcomes. Lastly, a positive psychology framework using ecological momentary assessment could be opted to see the effects on e.g., psychological wellbeing, since not all stress and ‘negative' emotions are necessarily a bad thing.

### Recommendations

All in all, biocueing could be a promising intervention to add to a digital treatment platform to help young people become more aware of their emotions. Be that as it may, notable improvements have to be made regarding the usability and acceptability of the smartwatch as well as more extensive integration of the smartwatch to a digital treatment platform before further implementing such an approach. Moreover, it is of importance to tailor the need to the individual of such an added intervention since large individual differences exist. The intervention might not work for all. It is of relevance to discover more about the contraindications before using a smartwatch. Finally, young people indicated that adding elements of gamification could improve learning and the use of the interventions.

## Data Availability Statement

The raw data supporting the conclusions of this article will be made available by the authors, without undue reservation.

## Ethics Statement

The studies involving human participants were reviewed and approved by METC, AMC: NL66345.018.18. The patients/participants provided their written informed consent to participate in this study.

## Author Contributions

MD, DN, TA, AP, MN, and MJ completed the initial study design. AM was involved with the execution of all phases of the study. DN, TA, MJ, AP, MN, and MA-J provided an expert assessment and feedback. The manuscript was written by MD, AM, and LN. All authors read and approved the final manuscript.

## Funding

Financial funding by The Netherlands Organisation for Health Research and Development for personnel, materials, and implementation (File number 60-63600-98-319).

## Conflict of Interest

The authors declare that the research was conducted in the absence of any commercial or financial relationships that could be construed as a potential conflict of interest.

## Publisher's Note

All claims expressed in this article are solely those of the authors and do not necessarily represent those of their affiliated organizations, or those of the publisher, the editors and the reviewers. Any product that may be evaluated in this article, or claim that may be made by its manufacturer, is not guaranteed or endorsed by the publisher.

## Footnotes

^1^All quotes were translated from Dutch to English.
