# Corrigendum: Usability, feasibility, and effect of a biocueing intervention in addition to a moderated digital social therapy-platform in young people with emerging mental health problems: a mixed-method approach

**DOI:** 10.3389/fpsyt.2024.1364965

**Published:** 2024-02-07

**Authors:** Marilon van Doorn, Laurens A. Nijhuis, Anne Monsanto, Thérèse van Amelsvoort, Arne Popma, Monique W. M. Jaspers, Matthijs L. Noordzij, Ferko G. Öry, Mario Alvarez-Jimenez, Dorien H. Nieman

**Affiliations:** ^1^ Amsterdam University Medical Centers, Amsterdam, Netherlands; ^2^ Department of Psychiatry and Neuropsychology, Maastricht University, Maastricht, Netherlands; ^3^ Department of Medical Informatics, Amsterdam Public Health Research Institute, Amsterdam UMC-Location AMC, University of Amsterdam, Amsterdam, Netherlands; ^4^ Department of Psychology, Health and Technology, University of Twente, Enschede, Netherlands; ^5^ Buurtzorg Jong, Almelo, Netherlands; ^6^ Centre for Youth Mental Health, The University of Melbourne, Parkville, VIC, Australia; ^7^ Orygen, Parkville, VIC, Australia

**Keywords:** biocueing, indicative prevention, stress- and emotion regulation, youth mental health, e-health, early detection and intervention

**Text Correction**

In the published article, there was an error in the text. The participant numbers in the quotes are incorrect, namely ‘P47’ and ‘P83’, these should be ‘P2’ and ‘P5’ respectively.

A correction has been made to **Results**, ***Phase 3-Evaluating the Intervention With End Users***. The corrected sentences appear below:

“[P2]: “Well, I haven’t thought about it [using the ENYOY-platform], and often when it [the watch] vibrated, I wasn’t near my laptop. I like it better to use it on my laptop than to use it on my phone. I didn’t open my laptop the past week, so that must’ve played a role.”

[P5]: “I used it [the ENYOY-platform] once or twice, because this phone wasn’t connected to the internet, which made it more of an effort, because then I had to see when I receive a notification and ask myself “why, when?” and then I have to use the platform here [in the bedroom] on my laptop. So for me, it’s a bit of an obstacle, because there are more actions involved.”

The authors apologize for this error and state that this does not change the scientific conclusions of the article in any way. The original article has been updated.

