# Supplementary material for: Usability, Feasibility, and Effect of a Biocueing Intervention in Addition to a Moderated Digital Social Therapy-Platform in Young People With Emerging Mental Health Problems: A Mixed-Method Approach
Source: Front Psychiatry. 2022 May 25;13:871813. doi: 10.3389/fpsyt.2022.871813 (PMC9174529; doi:10.3389/fpsyt.2022.871813)
Supplement: Supplementary file 1 [file Data_Sheet_1.DOCX]

**Appendix A.** Results of the thematic analysis in Phase 1 - *Understanding and specifying end user needs regarding the interventions*

The following relevant themes were subtracted from the data of the focus group: Perceived use of the intervention for peers, functionality of the Sense-IT/smartwatch, functionality of the platform, and obstacles for use. These four themes will be explored consecutively in the next section.

## *Perceived use of the intervention*

All participants indicated that using the intervention would be useful to them, and most mentioned that it would have been helpful for them in the past to be able to recognize stressful situations, see patterns in their behaviour and be more equipped to deal with them. One participant mentioned that young people often forget to use the platform, and thought that getting notifications on the Sense-IT when stressed may remind young people to use the platform to act on their mental health. Most of the participants mentioned the value of the Sense-IT in terms of becoming aware of emotions and bodily sensations.

Participant 3*: I used to not even notice that [my] heartbeat was beating faster, that I had sweaty palms or that my face was turning red, [...], even that I didn’t notice. I didn’t feel anything in my body [...]. I really needed other people to say to me ‘I think you have sweaty palms’ [...] or those kind of things ^^[[1]](#footnote-1)^^.*

However, another participant indicated being too aware of internal bodily sensations and was prone to interpreting this as “panic”. Both participants mentioned that becoming aware of their emotions would be a useful way of learning to cope with them, however the awareness of emotions and bodily sensations of young people should be addressed and explained before using the Sense IT in order to tailor the Sense IT to individual’s needs.

*Functionality of the Sense-IT/smartwatch*

One of the participants mentioned that when one becomes stressed, it might be especially challenging to take action. Therefore, the panel suggested that the steps to log on to the platform are reduced to a minimum, for example by providing a direct link from the smartwatch to the platform, or exporting exercises from the platform to the phone connected to the smartwatch. Additionally, other participants mentioned that to enhance emotion recognition, it may be useful to add optional exercises on the platform regarding emotion recognition.

Participant 2: ‘*I was thinking about being aware of emotions… It might be challenging to be able to do that in a stressful moment [...]. Maybe you just notice panic, and nothing else. It might help to provide exercises that help with that, for example an ABC-scheme^^[[2]](#footnote-2)^^. Maybe that is asking a lot, but then you can reflect, in that moment, on; what am I actually feeling right now?’*

Further, most participants opted to add questions to the smartwatch that could be useful to ask oneself when feeling stressed. E.g. ‘What am I doing at this moment?’, ‘who am I with?’ or ‘what do I need/what could help me?’.

Furthermore, there was no consensus about adding notifications for a *low* heartbeat. It was mentioned that it could be useful to also focus on positive interactions with the Sense-IT, it might be confusing to receive signals for both a low and high heartbeat, and it could work if the notification were to be different.

## *Functionality of the platform*

A critique mentioned by a participant was that the platform is not equipped for ad hoc exercises when stress-levels are already high. It is recommended that additional exercises that meet this demand are added to the platform. Another participant mentioned that they have experienced difficulties in differentiating between emotions, and suggested exercises that improve emotional literacy and awareness.

Participant 2: *‘[...] I think that’s step one, an exercise to help with emotional differentiation. I always use an emotional wheel ^^[[3]](#footnote-3)^^, for myself, that I made while I was in therapy.*

There was consensus among participants that the toolkit on the ENYOY-platform is a useful way to quickly access exercises that have helped in the past when feeling stressed. They noted that it is even an option to personalize categories in which exercises are saved (e.g. anger, sadness) to quickly access the right exercise. Moreover, it was advised to emphasize that the platform works well on smart devices since these might make it more accessible for young people.

*Obstacles for use*

Participants mentioned the aesthetics of the smartwatch as a factor that could make them less inclined to use it, indicating the smartwatch should fit a young person’s personality and lifestyle to ensure usability and benefit of the intervention. Additionally, discretion was mentioned as a barrier in intervention usage. They wondered whether other people would notice the buzzing of the smartwatch, and felt they would experience shame if other people would figure out what the smartwatch was used for. Third, creating and adhering to new habits, such as charging an additional smartphone and smartwatch every night, was mentioned as a potential challenge for users. Fourth, one participant mentioned that they were not able to use their smartwatch due to working standards imposed by employers. Therefore, additional support in creating new habits at the start of the intervention should be indicated. Sixth, a participant mentioned they were already extremely aware of their bodily sensations, and stated that drawing more attention to these sensations might cause even more distress, rendering the intervention counterproductive. Therefore, high interoceptive awareness might have to be considered as a contraindication for the smartwatch intervention or at least be closely monitored to prevent participants from quitting.

*Participant 1: ‘Something else that crossed my mind [...], I was very aware of my bodily sensations, and I think that if a watch would’ve increased this awareness and that would not be helping for me.’*

All participants indicated that a user manual for the Sense-IT should be provided and that the platform use should be personalized to highlight features that have added value for users using the smartwatch (e.g., the toolkit and the ability to label exercises with tags).

*Participant 1: ‘I think you have to provide a clear explanation, more than just what ENYOY has to offer [...], so someone sees the utility and how it can be of added value to them personally.’*

1. All quotes were translated from Dutch to English [↑](#footnote-ref-1)
2. ABC scheme is a commonly used technique in Cognitive Behavioral Therapy used to indicate Activating events, Beliefs and Consequences in the form of emotions and behaviors (Beck, 2011) [↑](#footnote-ref-2)
3. The emotion wheel is a circle with 40 distinctive emotion labels, used to learn to recognize and label emotions (77. Shuman V, Schlegel, K., and, Scherer, K. Geneva Emotion Wheel Rating Study. Geneva: University of Geneva, Swiss Center for Affective Sciences; 2015. p. 1-13) [↑](#footnote-ref-3)
